# Quality of Life Assessment of Breast Cancer Patients Undergoing Chemotherapy in Jordan: A Cross-Sectional Study

**DOI:** 10.1155/ijbc/9936131

**Published:** 2025-03-01

**Authors:** Sijood Janabi, Lobna Gharaibeh, Ibrahim Aldeeb, Ali Abuhaliema

**Affiliations:** ^1^Biopharmaceutics and Clinical Pharmacy Department, Al-Ahliyya Amman University, Amman, Jordan; ^2^Faculty of Pharmacy, Zarqa University, Zarqa, Jordan

**Keywords:** assessment, breast cancer, chemotherapy, Jordan, quality of life

## Abstract

**Purpose:** Breast cancer patients are subjected to many hardships during chemotherapy which negatively affects the patient's quality of life. The current study was conducted to identify aspects with low scores and produce untoward effects on the quality of life.

**Results:** The results of the study showed that the social functioning domain achieved high quality of life mean score of 76.68 ± 32.94 while emotional functioning attained 38.18 ± 29.61. The most apparent symptoms detected were insomnia and fatigue followed by pain and loss of appetite. Regarding EORTC-BR45, higher score and better quality of life were observed in the body image domain with a mean score of 60.72 ± 37.19, while the future perspective domain achieved low quality of life of 35.41 ± 42.9, and the most obvious symptom for patients was upset by hair loss.

**Conclusion:** The results of the study showed the impact of chemotherapy on the lives of patients and highlighted the aspects that need greater focus by healthcare providers in Jordan. In addition to providing treatment, emotional and psychological support are necessary to improve the quality of life for these women.

## 1. Introduction

In Jordan, breast cancer is the most common cancer among women, and it is the third cause of death after lung and colorectal cancer [1]. Quality of life (QoL) is a concept for individuals or population related to both positive and negative elements in all parts of life such as health (physical and mental), education, work environment, relationships, and social status [2]. Recently, patients' QoL got more attention in decision-making and treatment choices. QoL of breast cancer patients is affected by various factors such as social and economic status, surgery, body image during and after treatment, and many other psychological factors [3].

Breast cancer is a life-threatening disease, and it has major negative effects on patients' lives. The disease results in body image problems because of surgery and chemoradiotherapy. In addition, financial struggles, adverse effects of treatment, and stress lead to a decrease in patient's QoL [4].

QoL of breast cancer patients was studied extensively worldwide, and many aspects of QoL have improved, but some still have a negative impact on patients' family, social life, body image, and sexual performance [5]. A meta-analysis that evaluated the QoL of 9012 patients with breast cancer using the EORTC QLQ-C30 revealed that women who were currently under treatment had lower scores than those who completed treatment. This suggests that chemotherapy poses serious negative consequences on the lives of women which must be taken into consideration [6]. Kapoor et al. study showed that the area of residence and whether the patient had surgery or not affected their QoL. Patients who had surgery obtained better scores in their future prospectives and sexual enjoyment. Additionally, women residing in urban areas had high scores in both scales which might indicate problems inequity of the distribution of therapeutic management and support for breast cancer survival that is reflected on QoL [7]. Similarly, Khazi et al.'s findings showed that patients who underwent breast surgery achieved a better QoL [8].

A recent review conducted by Heidary et al. examined thoroughly qualitative studies on the QoL of women with breast cancer. Physical, spiritual, and psychological concerns were the most frequent. Ethnic and racial differences in different geographical regions dictate variations in concerns among women [9]. Therefore, it is necessary to assess the QoL in breast cancer survivors in Jordan to take into consideration disparities among different cultures.

In Jordan, health equity for cancer patients is a great issue since services provided in various national medical institutions vary. Jordan does not have a national cancer survivorship program. King Hussein Cancer Centre (KHCC) is the only institution with a breast cancer survivorship program that focuses on clinical care including all survivorship dimensions. This type of care is lacking for a great number of patients who receive cancer therapy elsewhere. In addition, follow-up by the primary care physicians is not pursued for most patients which puts huge strain on the oncology departments and specialists. Moreover, support, psychological assessment, and care are not priorities, and patients are left to manage the detrimental effects of the disease and chemotherapy without adequate support [10].

The current work, which is the first in Jordan, aims to evaluate the QoL of breast cancer patients on chemotherapy using the European Organization for Research and Treatment of Cancer Core Quality of Life Questionnaire (EORTC QLQ-C30) and the European Organization for Research and Treatment of Cancer Quality of Life Questionnaire Updated Breast Cancer Module (EORTC QLQ-BR45). Findings from this study will help improve the management of side effects of drugs and emphasize on issues that are troublesome to women on chemotherapy.

## 2. Methodology

This was a cross-sectional study conducted at Al-Basher Hospital in Amman in the oncology department from February 2023 to July 2023. Information was collected from patients by face-to-face interviews using two validated questionnaires, EORTC QLQ-BR45 and EORTC QLQ-C30, to evaluate the QoL of breast cancer patients on chemotherapy. At the start of the meeting, the interviewer introduced herself, briefly talked about the purpose of the study, and asked if the patients would like to enroll in the study. The duration of each interview ranged from 15–20 min depending on patient's ability to answer the questions and their overall health condition on the day of the interview.

The ethical approval (number MOH/REC/2023/32) was obtained on January 25, 2023, from the ethics committee of the Ministry of Health prior to commencing the recruitment. Before starting the interview, each patient signed an informed consent form that guarantees anonymity of information and that the data will be used for scientific research purposes and publication. Data collected and generated by this research study is considered strictly confidential by the investigators. Each patient is only identified by a unique code number.

Inclusion criteria include females 18 years or older with breast cancer who are on chemotherapy for at least 4 weeks, regardless of having surgery, metastasis, or radiotherapy. Patients who received medication other than chemotherapy, such as hormonal or biological therapy, and patients whose information was not complete were excluded from the study.

## 3. Questionnaires

The QLQ-C30 questionnaire is a core questionnaire that is used to assess the QoL among any type of cancer patient composed of 30 items to assess QoL in different parameters including physical, psychological, and social issues. The Breast Cancer module QLQ-BR45 is a supplementary questionnaire used in conjunction with the QLQ-C30 to assess body image, sexual functioning, breast satisfaction, systemic therapy side effects, arm symptoms, breast symptoms, endocrine therapy symptoms, skin mycosis symptoms, and endocrine sexual symptoms. In addition, single items assess sexual enjoyment, future perspective, and being upset by hair loss.

Both questionnaires are originally written in the English language. In this study, the questionnaires were translated into the Arabic language by a translation specialist and reviewed by two field experts to match the Arabic with the English versions. The Arabic version of EORTC QLQ-BR45 and EORTC QLQ-C30 was previously validated and used in research [11, 12]. The length of the questionnaires and the suitability of questions were assessed in the first five interviews and were not included in the analysis of data.

The scores of both questionnaires ranged from 0 to 100 and were calculated manually using specific equations. A high score for a functional scale and global health scale represents a high/healthy level of functioning and represents a high QoL, but a high score for a symptom scale represents a high level of symptomatology and problems.

Patients were divided into two groups depending on their scores; scores ≤ 33 for the functional scales and the global QoL were considered inadequate, and scores ≥ 66 were considered adequate. For the symptom scale, the score is reversed, scores ≤ 33 were considered adequate, and those ≥ 66 were considered inadequate [12, 13].

## 4. Statistical Analysis

The statistical analysis of the study data was carried out using the Statistical Package for Social Sciences (IBM SPSS for Windows, Version 20) software. Continuous data was presented as Mean ± SD, and categorical data was presented as frequency (percent). A comparison of the means of two independent groups was carried out using an independent sample *t*-test to determine whether there is statistical evidence. Comparison between more than two independent categories was analyzed using a one-way analysis of variance (ANOVA). Possible predictors of QoL scores were assessed using univariate and multivariate linear regression. *p* values < 0.05 are considered statistically significant.

## 5. Results

A total of 124 patients were approached for the interview, and 120 patients agreed to participate (96%). Twenty-five patients were excluded from the study because of missing data. The final number of participants with full information and complete data was 95 women.

**Figure 1:**
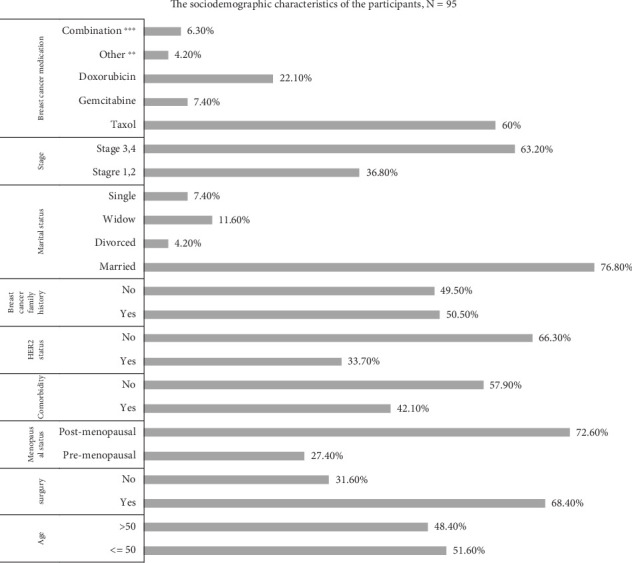
The sociodemographic and clinical characteristics of the participants, *N* = 95. ⁣^∗∗∗^Only 6% of the participants used combination therapy that included TCHP (docetaxel, carboplatin, trastuzumab, and pertuzumab) and AC (doxorubicin and cyclophosphamide). TC (Taxotere and cyclophosphamide). ⁣^∗∗^Two patients received vinorelbine chemotherapy, one patient received carboplatin, one patient received lapatinib chemotherapy, and they were all included in the category “others.”

Almost 50% of the participants were older than 50 years of age. More than two-thirds were in advanced stages, and the most frequently used chemotherapeutic agent was Taxol. The sociodemographic and clinical characteristics of the participants are shown in Figure 1.

QoL assessment revealed that in terms of global health, which reflects general health and QoL in general, 17 of the participants had inadequate scores. In the functional scale, half the women had inadequate emotional scores, and it was the highest among the functional scale which suggests that emotional aspects of the women's QoL are highly affected. In the symptom scale, insomnia was the most troubling symptom with 48.4% with inadequate scores (Table 1).

Assessment using QLQ-BR45 showed that almost two-thirds of the participants had an inadequate functional score on the future perspective which was the highest among the attributes of the functional scale. Among the symptom scale being upset because of losing hair had the highest percentage of inadequate score (Table 1).

Global health score was significantly higher for premenopausal women and those in Stage 1 or 2 compared to postmenopausal women and those in later stages of cancer. Role functioning scores for participants in early stages (1 or 2) were higher compared to those in later stages (3 or 4). Additionally, participants on combination medications had higher role functioning scores compared to those on a single drug or on other medications. Women older than 50 had significantly higher emotional functioning scores, so did women with positive HER2 status. In the cognitive functioning score, women older than 50 had a higher score compared to younger women (Table 2).

In the symptom scale, insomnia was higher in women younger than 50 compared to older women, fatigue was higher in the other regimens compared to a combination of Taxol, gemcitabine, and doxorubicin. Pain score was higher in women with late stages compared to early stages. Similarly, the diarrhea score was higher in women in the late stages of breast cancer, and financial difficulties were higher for patients who had surgery, Table 2.

In the QLQ-BR45, older women had higher body image and future perspective scores compared to younger women. Women on gemcitabine had the highest score on arm symptoms compared to other regimens, divorced and single women had the lowest score on being upset by hair loss (Table 3).

Possible predictors of functional scale score were assessed using multivariate linear regression analysis. Predictors of low role functioning score were postmenopausal (*B* = −0.247) and having later stages of breast cancer (*B* = −0.226). The predictor of lower social functioning was being divorced compared to being a single woman (*B* = −0.356) (Supporting Information Table S4).

## 6. Discussion

In Jordan, cancer treatment is based mainly on chemotherapy, and the focus of healthcare measures is based on the survival of the patient. However, this is not enough to lead a healthy, fruitful, and fulfilling life postbreast cancer diagnosis. The different aspects of the QoL are an important indicator of the ability of women to regain or maintain the quality of their lives and shed light on areas that need attention. Unfortunately, the emotional, functional, and social needs of women who are on chemotherapy are underestimated in favor of treating the side effects of chemotherapy. This is the first study to quantitatively assess the QoL of the breast cancer patients in Jordan based on validated questionnaires.

Regarding the QoL of breast cancer survivors using EORTC QLQ-C30, the social functioning domain achieved high QoL while emotional functioning achieved a low QoL mean score. This finding is supported by many studies that reported functional scales of the QLQ-C30, where the majority of subjects had scores indicating good social functioning; the lowest scores were noted for physical functioning and role functioning, while the highest scores were for cognitive and social functioning [14–16]. The high scores of social functioning might indicate good social support from the community, where tight bonds between the breast cancer survivor and her close friends, neighbors, and family members provide social support. However, the low emotional scale should be addressed by providing psychological guidance and therapy to breast cancer survivors through support groups, psychiatric sessions, and consultation [17]. A study from Saudi Arabia showed that emotional function is an important aspect, which is directly related to the patients' satisfaction among breast cancer patients in palliative care [18].

The most apparent symptoms detected in this study were insomnia and fatigue followed by pain and loss of appetite. These findings are in agreement with a study from Kuwait on 348 patients, which reported poor to average functioning; only 5.8%–11.2% had scores that met the ≤ 33% criterion for problematic functioning, while 12.0%–40.0% met the > 66% criterion for more severe symptoms. Most of the patients (47.8%–70.1%) met the > 66% criterion for “good functioning” on the BR-23 functional scales [19]. Concerning the EORTC QLQ -BR45 symptom scale, the hair loss item was a major concern. Similarly, Jassim and Whitford found that hair loss was the leading side effect that affected the QoL followed by arm symptoms [19].

Comparison of variables in global health and functional scale in QLQ-C30 revealed that there was a statistical association/correlation between emotional, cognitive functioning, and age. Patients older than 50 had better emotional and cognitive scores. This might be explained by assuming that older patients are wiser, have more life experience, and are well-seasoned, which endows them with emotional stability and better understanding of hardships.

The present study showed a statistically significant difference between global health, role functioning, and stage of the disease. Similarly, Timperi et al.'s study among Vietnamese breast cancer women showed that the breast cancer stage of the studied sample was associated with and negatively affected global health and role functioning [20]. Later stages of disease are more stressful and advanced and affect more aggressively the general health or well-being of the patient. Other studies also reported that QoL among breast cancer patients was affected by their breast cancer stage [21, 22].

A statistically significant association was found between medication type and role function. Patients who received combination drugs had better role function, and a possible reason is that combination drugs had better efficacy and treatment outcomes that led to better Qol. Additionally, global health was associated with menopausal status. Postmenopausal patients had lower global health scores than premenopausal patients, which might be due to the additional postmenopausal symptoms that women have to endure in addition to chemotherapy side effects. The general well-being of women in our study with no comorbidities was higher than those with comorbidities. Strangely enough, one study revealed that physical well-being and other QoL domains were positively related to the number of comorbidities [20].

Patients younger than 50 suffered more from insomnia. This finding is supported by Beverly et al.'s study that found an association between insomnia and breast cancer patients who received chemotherapy [23]. Regarding medications, patients who consumed carboplatin, lapatinib, or vinorelbine experienced fatigue and pain which is supported by a case report of women who received carboplatin and suffered from fatigue [24].

Older patients with comorbidities had a problem regarding their body image and future perspective. Davis et al. suggest that body image is considered important in older breast cancer patients and may impact or be impacted by several factors including age [25]. Moreover, patients with comorbidities believe they are at high risk for being sick and have a little ability to fight cancer which affects their future perspectives [26].

In the linear regression model, postmenopausal women achieved lower scores, in global health mean QoL than their counterparts—premenopausal women. This finding is similar to a study conducted in Sweden that found a decrease in the overall QoL of postmenopausal breast cancer patients after starting therapy [27]. This finding may be explained by postmenopausal symptoms that affect women at this age and may contribute to additional hardships. Moreover, the physiologic changes that women experience after menopause may contribute to this association. Another significant finding is the lower score for divorced patients and social functioning. This finding is supported by a study conducted in South Korea that showed that divorced breast cancer patients had lower QoL and social functioning than nondivorced patients [28]. Additionally, widowers had a higher score in role functioning compared to single women. Similar results were found in another study that found an association between marital status and QoL of breast cancer patients in favor of the married and widowed women [29]. Stages 3 and 4 predicted lower scores; this finding can be explained by the fact that later stages of disease affect negatively the patient's overall daily activities. Similarly, Hassen et al. showed that patients at Stage 3 have lower QoL [30]. Patients at later stages visit the healthcare facilities more frequently, and the burden of transportation and waiting causes limitations to normal daily life that affect the role function. Based on the previous discussion, the social status of the patients, menopause, and disease stage should always be taken into consideration during the journey of treatment since these variables predict inadequate QoL scores. Targeted management strategies should be developed based on prioritizing scarce psychological and clinical support to vulnerable patients who need it the most.

The QoL of breast cancer survivors is dynamic and multifaceted. Fortner et al. emphasized that QoL is not static, and patients have altered QoL at different disease stages, for instance, after diagnosis, before and after treatment, and long-term effects of cancer and treatment modalities [31]. It was observed that, if women were provided with social support after their treatment for breast cancer, it could help to decrease mortality and recurrence of cancer, especially during the early posttreatment phase [32]. Some treatment choices may have a positive impact on QoL. A study identified, contrary to common belief, significantly better QoL while using extended adjuvant endocrine therapy in breast cancer patients [33].

This is the first study to examine the QoL of breast cancer survivors on chemotherapy. Limitations of the study include small sample size and the inclusion of patients on chemotherapy rather than wider range of drugs which would have allowed comparison between different drug groups. In our study, the type of treatment did not have substantial effect on different scales. This might be explained by the low sample size and the fact that we included only chemotherapy which did not allow us to explore differences among a wider range of medications.

## 7. Conclusion

Breast cancer is the most prevalent type of cancer in Jordanian women. Treatment with chemotherapy can have tremendous effects on their QoL. Findings from this study revealed the substantial impact of chemotherapy on the lives of patients. The emotional status of women had the lowest score, and postmenopausal women had lower QoL scores compared to premenopausal women which justifies focused efforts on the emotional status of postmenopausal women. In alignment with chemotherapy and surgery to save the lives of breast cancer patients, efforts should be implemented to improve QoL and enable women to continue healthy, productive, and satisfactory lives.

## Figures and Tables

**Table 1:** Assessment of quality of life of breast cancer survivors using EROTIC QLQ-C30 and EROTIC QLQ-BR45.

**Scale**	**N**	**Number of items**	**M** **e** **a** **n** ± **SD**	**N** ** (%) ** **s** **c** **o** **r** **i** **n** **g** < 33.3^**a**^	**N** ** (%) ** **s** **c** **o** **r** **i** **n** **g** > 66.7^**a**^
Global Health Scale	95	2	95.59 ± 19.01	17 (17.9%)	52 (54.7%)
*Functional scale* ^b^					
Physical functioning	95	5	58.65 ± 23.18	10 (10.5%)	46 (48.4%)
Role functioning	95	2	65.77 ± 34.58	33 (34.7%)	60 (63.2%)
Emotional functioning	95	4	38.18 ± 29.61	53 (55.84%)	24 (25.3%)
Cognitive functioning	95	2	66.80 ± 30.43	26 (27.4%)	58 (61.1%)
Social functioning	95	2	76.68 ± 32.94	21 (22.1%)	73 (76.8%)
*Symptoms scale* ^c^					
Fatigue	95	3	44.52 ± 32.65	46 (48.4%)	30 (31.6%)
Nausea and Vomiting	95	2	21.20 ± 30.46	79 (83.2%)	13 (13.7%)
Pain	95	2	38.07 ± 29.17	26 (65.3%)	27 (28.4%)
Dyspnea	95	1	25.20 ± 31.05	77 (81.1%)	18 (18.9%)
Insomnia	95	1	48.07 ± 38.27	49 (51.6%)	46 (48.4%)
Appetite loss	95	1	29.46 ± 35.07	68 (71.6%)	27 (28.4%)
Constipation	95	1	25.60 ± 32.83	72 (75.8%)	23 (24.2%)
Diarrhea	95	1	16.11 ± 23.77	83 (87.4%)	12 (12.6%)
Financial difficulties	95	1	30.88 ± 39.32	65 (68.48%)	30 (31.6%)
*EROTIC QLQ-BR45*					
*Functional scale* ^b^					
Body image	95	4	60.72 ± 37.19	33 (34.7%)	54 (56.8%)
Future perspective	95	1	35.41 ± 42.91	64 (67.4%)	31 (32.6%)
Sexual functioning	95	2	75.34 ± 29.79	9 (9.5%)	34 (35.8%)
Sexual enjoyment (for married only)	95	1	77.81 ± 31.04	9 (9.5%)	39 (41.1%)
Breast satisfying (for surgery only)	95	2	59.72 ± 32.36	27 (28.4%)	37 (38.9%)
Symptoms scale^c^					
Systemic therapy side effects	95	7	44.77 ± 21.06	38 (40%)	18 (18.9%)
Upset by hair loss	95	1	66.31 ± 46.00	32 (33.7%)	63 (66.3%)
Arm symptoms	95	3	32.48 ± 23.25	62 (65.3%)	11 (11.6%)
Breast symptoms	95	4	14.37 ± 16.09	88 (92.6%)	3 (3.2%)
Endocrine therapy symptoms	95	10	42.74 ± 20.95	32 (33.7%)	17 (17.9%)
Skin mucosis symptoms	95	6	32.05 ± 18.83	59 (62.1%)	7 (7.4%)
Endocrine sexual symptoms	95	4	35.98 ± 34.00	57 (60%)	31 (32.6%)

^a^For functional scale, subjects scoring < 33.3% have problems; those scoring > 66.7% have good functioning. For the symptom scale, subjects scoring < 33.3% have good functioning; those scoring > 66.7% have problems.

^b^For the functional scale, a higher score indicates better functioning.

^c^For the symptom score, a higher score indicates worse functioning.

**Table 2:** Comparison of variables in global health, functional scale, and symptomatic scale in QLQ-C30 (using independent sample *t* test for two categories and one-way ANOVA for more than two categories).

**Variable**	**Global health**		**Functional scale**						
	**Global health mean (SD)**		**Physical functioning mean (SD)**	**Role functioning mean (SD)**		**Emotional functioning mean (SD)**	**Cognitive functioning mean (SD)**		**Social functioning mean (SD)**
*Age*									
≤ 50	60.5 (19.6)		59.28 (23.08)	65.26 (35.57)		30.91 (29.04)	60.48 (29.84)		71.81 (36.51)
> 50	58.7 (18.5)		57.97 (23.51)	66.30 (33.78)		45.91 (28.51)	73.52 (29.90)		81.86 (28.13)
*p* value	0.65		0.78	0.88		**0.013**	**0.036**		0.13
*Surgery*									
Yes	58.73 (19.80)		57.32 (24.18)	65.86 (35.25)		36.56 (28.36)	67.63 (30.21)		76.16 (33.22)
No	61.43 (17.34)		61.53 (20.93)	65.56 (33.66)		41.66 (32.36)	65.00 (31.33)		77.80 (32.85)
*p* value	0.52		0.41	0.96		0.43	0.70		0.82
*Menopausal status*									
Pre	67.03 (18.95)		64.07 (21.11)	76.30 (32.05)		35.23 (34.06)	58.26 (29.21)		69.92 (37.40)
Post	56.78 (18.39)		56.60 (23.73)	61.79 (34.87)		39.28 (27.94)	70.01 (30.46)		79.23 (31.01)
*p* value	**0.018**		0.16	0.068		0.55	0.09		0.22
*Comorbidity*									
Yes	56.67 (19.60)		54.62 (22.16)	59.55 (35.67)		39.80 (26.29)	67.90 (32.55)		75.05 (33.52)
No	61.70 (18.46)		61.58 (23.65)	70.29 (33.35)		37.00 (31.99)	66.00 (29.08)		77.87 (32.76)
*p* value	0.20		0.15	0.13		0.65	0.76		0.68
*HER2 status*									
Yes	65.34 (20.49)		62.53 (26.63)	75.53 (35.93)		47.40 (31.17)	64.56 (31.88)		84.87 (27.93)
No	56.66 (17.67)		56.68 (21.17)	60.80 (33.05)		33.49 (27.87)	67.93 (29.85)		72.52 (34.68)
*p* value	**0.035**		0.24	0.058		**0.03**	0.62		0.084
*Breast cancer family history*									
Yes	58.04 (19.17)		56.77 (23.82)	59.33 (33.93)		35.27 (28.10)	63.85 (33.55)		72.93 (36.16)
No	61.17 (18.91)		60.57 (22.59)	72.34 (34.33)		41.14 ()31.09	69.80 (26.88)		80.51 (29.18)
*p* value	0.426		0.427	0.067		0.336	0.343		0.265
*Marital status*									
Married	58.58 (18.58)		58.24 (24.26)	64.13 (35.18)		38.24 (29.52)	66.39 (30.36)		76.97 (32.59)
Divorced	41.75 (1.56)		43.25 (33.78)	45.75 (41.70)		25.00 (24.68)	62.50 (47.87)		20.75 (24.94)
Widow	68.18 (20.29)		65.45 (14.56)	89.45 (18.58)		44.81 (33.07)	72.72 (25.04)		89.36 (21.50)
Single	66.71 (3.47)		61.00 (13.15)	57.00 (31.89)		34.57 (30.62)	64.14 (34.07)		85.71 (26.29)
*p* value	0.071		0.428	0.066		0.700	0.908		**0.002**
*Stage*									
Stages 1 and 2	65.74 (19.74)		64.02 (16.13)	79.54 (28.07)		41.28 (30.53)	67.60 (28.55)		75.25 (30.38)
Stages 3 and 4	56.00 (17.77)		55.51 (26.05)	57.73 (35.67)		36.36 (29.16)	66.33 (31.69)		77.51 (34.56)
*p* value	**0.019**		0.084	**0.003**		0.438	0.846		0.749
*Breast cancer medication*									
Taxol	57.61 (21.41)		56.01 (25.00)	63.54 (34.91)		39.92 (30.32)	68.10 (31.05)		71.07 (35.32)
Gemcitianib	52.42 (11.73)		45.57 (22.24)	37.85 (30.03)		38.00 (23.00)	83.42 (31.76)		85.71 (26.29)
Doxorubicin	66.38 (12.45)		71.71 (15.17)	79.33 (32.53)		32.14 (32.06)	60.19 (26.70)		80.95 (32.18)
Others	54.00 (20.92)		45.25 (21.26)	41.50 (17.00)		33.50 (9.81)	66.50 (38.68)		91.75 (16.50)
Combination	66.66 (14.76)		62.61 (10.74)	89.00 (17.04)		46.00 (33.15)	58.33 (31.11)		94.50 (13.47)
*p* value	0.244		**0.022**	**0.010**		0.817	0.462		0.277

**Symptomatic scale**									
**Variable**	**Fatigue** **Mean (SD)**	**Nausea and vomiting** **Mean (SD)**	**Pain** **Mean (SD)**	**Dyspnea** **Mean (SD)**	**Insomnia** **Mean (SD)**	**Appetite loss** **Mean (SD)**	**Constipation** **Mean (SD)**	**Diarrhea** **Mean (SD)**	**Financial difficulties** **Mean (SD)**
*Age*									
≤ 50	49.36 (32.50)	22.77 (34.61)	40.48 (28.59)	27.81 (34.27)	55.81 (36.95)	30.59 (35.94)	25.83 (30.70)	14.91 (22.64)	35.38 (38.79)
> 50	39.34 (32.35)	19.52 (25.58)	35.50 (29.87)	22.41 (27.30)	39.82 (38.30)	28.26 (34.46)	25.34 (35.30)	17.36 (25.13)	26.08 (39.72)
*p* value	0.136	0.606	0.408	0.400	**0.041**	0.748	0.943	0.618	0.252
*Surgery*									
Yes	44.06 (34.27)	20.50 (32.75)	39.52 (31.46)	22.50 (31.25)	49.21 (38.30)	30.26 (35.77)	25.10 (33.90)	16.35 (22.92)	36.43 (40.75)
No	45.50 (29.35)	22.70 (25.24)	34.93 (23.63)	31.03 (30.29)	45.60 (38.71)	27.73 (34.05)	26.66 (30.91)	15.56 (25.95)	18.86 (33.55)
*p* value	0.843	0.746	0.479	0.215	0.673	0.746	0.831	0.882	**0.042**
*Menopausal status*									
Pre	46.11 (36.41)	15.38 (24.44)	34.61 (27.80)	22.96 (29.44)	53.84 (39.02)	19.15 (30.04)	14.03 (21.42)	15.34 (23.56)	34.57 (39.45)
Post	43.19 (31.38)	23.39 (32.33)	39.37 (29.76)	26.04 (31.80)	45.89 (38.03)	33.34 (36.22)	29.95 (35.37)	16.39 (24.03)	29.49 (39.46)
*p* value	0.771	0.256	0.481	0.669	0.378	0.079	**0.034**	0.850	0.577
*Comorbidity*									
Yes	50.55 (32.80)	18.25 (24.02)	44.12 (28.58)	22.45 (29.64)	48.30 (39.25)	26.60 (34.76)	27.47 (34.54)	17.47 (25.07)	29.15 (39.38)
No	40.12 (32.12)	23.34 (34.46)	33.67 (29.06)	27.20 (32.15)	47.90 (37.89)	31.54 (35.46)	24.23 (31.78)	15.10 (22.98)	32.14 (39.57)
*p* value	0.125	0.424	0.085	0.465	0.961	0.500	0.638	0.635	0.716
*HER2 status*									
Yes	34.28 (32.39)	15.09 (24.81)	30.25 (29.38)	16.59 (22.39)	36.43 (35.34)	23.96 (33.11)	25.00 (32.85)	16.65 (25.46)	31.25 (38.78)
No	49.71 (31.78)	24.30 (32.71)	42.04 (28.47)	29.57 (33.96)	35.98 (38.60)	32.25 (35.96)	25.90 (33.08)	15.82 (23.09)	30.69 (39.88)
*p* value	**0.031**	0.165	0.062	0.054	**0.034**	0.279	0.900	0.873	0.949
*Breast cancer family history*									
Yes	50.66 (32.02)	17.00 (25.82)	42.33 (28.30)	30.45 (32.15)	51.35 (38.33)	29.87 (37.24)	25.62 (31.71)	18.68 (23.74)	31.95 (37.06)
No	38.23 (32.41)	25.48 (34.31)	33.72 (29.70)	19.82 (29.24)	44.72 (38.32)	29.04 (33.11)	25.57 (34.28)	13.46 (23.78)	29.78 (41.86)
*p* value	0.063	0.176	0.151	0.095	0.401	0.909	0.994	0.287	0.789
*Marital status*									
Married	45.00 (33.52)	22.31 (31.52)	37.90 (29.08)	25.92 (31.57)	51.15 (37.78)	29.68 (35.01)	26.46 (33.36)	14.57 (22.92)	31.50 (39.67)
Divorced	64.00 (47.44)	4.25 (8.50)	62.50 (36.75)	41.75 (50.05)	50.00 (43.11)	25.00 (50.00)	16.50 (19.05)	24.75 (16.50)	50.25 (33.50)
Widow	35.36 (25.87)	16.72 (23.61)	25.72 (18.76)	15.00 (17.23)	45.45 (42.92)	18.09 (22.90)	12.09 (22.49)	18.18 (27.42)	36.36 (45.86)
Single	42.71 (23.61)	26.28 (37.06)	45.28 (34.37)	23.85 (31.84)	19.00 (26.26)	47.57 (42.47)	43.00 (41.88)	23.85 (31.84)	4.71 (12.47)
*p* value	0.514	0.629	0.157	0.504	0.207	0.383	0.244	0.653	0.232
*Stage*									
Stages 1 and 2	36.45 (31.22)	19.05 (28.62)	30.02 (25.41)	17.05 (24.71)	42.88 (33.99)	34.34 (35.78)	21.88 (31.29)	7.57 (16.28)	25.68 (37.14)
Stages 3 and 4	49.21 (32.79)	22.45 (31.65)	42.76 (30.38)	29.95 (33.49)	51.10 (40.51)	26.61 (43.63)	27.76 (33.76)	21.08 (26.08)	33.91 (40.52)
*p* value	0.066	0.603	**0.039**	0.05	0.315	0.303	0.403	**0.007**	0.328
*Breast cancer medications*									
Taxol	48.71 (34.57)	18.38 (28.41)	42.08 (30.48)	25.08 (31.07)	46.75 (38.31)	32.17 (36.77)	26.87 (33.64)	17.52 (25.34)	23.73 (39.12)
Gemcitianib	60.28 (26.51)	21.42 (36.88)	52.28 (24.56)	33.28 (38.53)	71.42 (40.54)	33.14 (33.33)	33.42 (33.50)	19.00 (26.26)	38.14 (48.82)
Doxorubicin	27.85 (19.13)	34.09 (35.90)	22.28 (16.87)	22.14 (24.37)	43.00 (36.84)	27.00 (32.78)	25.38 (31.52)	17.38 (22.64)	20.66 (35.76)
Others	64.00 (32.03)	20.75 (24.94)	62.50 (31.25)	41.50 (41.98)	66.50 (38.68)	33.25 (47.14)	33.25 (47.14)	8.25 (16.50)	33.50 (38.68)
Combination	31.50 (38.84)	2.83 (6.94)	22.33 (29.27)	16.66 (40.82)	38.83 (39.03)	5.50 (13.47)	0.00 (0.00)	0.00 (0.00)	38.83 (49.07)
*p* value	**0.03**	0.168	**0.007**	0.701	0.369	0.506	0.366	0.477	0.735

*Note:* Bold *p* values are statistically significant.

**Table 3:** Comparison of variables in functional scale and symptomatic scale in QLQ-BR45 (using independent sample *t* test for two categories and one-way ANOVA for more than two categories).

**Variables**	**Functional scale**						
	**Body image**	**Future perspective**		**Sexual functioning**	**Sexual enjoyment** ^**b**^		**Breast satisfaction** ^**a**^
*Age*							
≤ 50	52.75 (37.77)	23.10 (38.01)		73.14 (30.93)	75.97 (30.49)		60.48 (30.63)
> 50	69.19 (35.01)	48.52 (44.31)		81.94 (26.14)	83.33 (33.36)		58.83 (34.77)
*p* value	**0.031**	**0.003**		0.381	0.508		0.839
*Surgery*							
Yes	56.15 (39.73)	30.72 (41.38)		77.08 (30.18)	77.09 (32.22)		
No	70.60 (29.20)	45.56 (45.07)		71.87 (29.63)	79.25 (29.49)		
*p* value	0.078	0.118		0.573	0.823		
*Menopausal status*							
Pre	55.80 (36.05)	23.07 (38.63)		72.84 (27.30)	77.26 (25.01)		64.56 (34.93)
Post	62.56 (37.70)	40.05 (3.77)		76.98 (31.76)	78.17 (34.84)		58.14 (1.69)
*p* value	0.433	0.085		0.642	0.922		0.495
*Comorbidity*							
Yes	69.37 (37.76)	45.82 (45.12)		76.54 (30.58)	82.41 (29.13)		58.20 (35.89)
No	54.41 (35.81)	27.83 (39.94)		74.68 (29.83)	75.29 (32.21)		60.61 (30.53)
*p* value	0.052	**0.043**		0.840	0.453		0.775
*HER2 status*							
Yes	64.03 (36.45)	42.68 (45.79)		73.12 (31.94)	75.94 (35.83)		39.66 (27.74)
No	59.03 (37.74)	31.71 (41.24)		76.67 (28.89)	78.93 (28.37)		69.29 (30.17)
*p* value	0.539	0.241		0.694	0.751		0.001
*Breast cancer family history*							
Yes	63.91 (35.37)	44.41 (44.23)		76.10 (29.63)	80.00 (29.87)		61.77 (34.84)
No	57.44 (39.08)	26.21 (39.89)		74.09 (30.86)	74.16 (33.44)		57.85 (30.32)
*p* value	0.400	**0.038**		0.824	0.547		0.629
*Marital status*							
Married	58.46 (37.43)	34.68 (43.21)		34.68 (43.20)			59.64 (31.52)
Divorce	52.00 (44.33)	16.50 (19.05)		16.50 (19.05)			33.25 (27.35)
Widow	65.18 (34.36)	45.45 (47.80)		45.45 (47.80)			80.00 (29.90)
Single	82.14 (34.93)	38.00 (44.85)		38.00 (44.85)			61.16 (39.03)
*p* value	0.402	0.706		0.706			0.200
*Stages*							
Stages 1 and 2	59.08 (37.02)	25.68 (38.85)		64.48 (31.39)	71.20 (30.55)		61.07 (34.03)
Stages 3 and 4	61.66 (37.57)	41.08 (44.43)		80.28 (28.13)	80.81 (31.25)		58.76 (31.54)
*p* value	0.745	0.092		0.108	0.325		0.779
*Breast cancer medications*							
Taxol	63.47 (37.68)	42.07 (43.90)		71.64 (32.62)	75.33 (35.34)		61.08 (34.58)
Gemcitabine	65.42 (44.24)	23.85 (41.84)		83.33 (27.96)	83.33 (27.96)		72.16 (32.92)
Doxorubicin	53.19 (35.83)	30.14 (43.35)		78.30 (26.15)	80.10 (23.29)		50.00 (30.53)
Others	62.50 (28.59)	33.25 (47.14)		91.50 (12.02)	83.50 (23.33)		66.75 (27.35)
Combination	54.16 (41.47)	5.50 (13.47)		72.11 (34.88)	77.66 (38.68)		55.50 (24.43)
*p* value	0.836	0.277		0.828	0.978		0.672

**Symptomatic scale**							
**Variables**	**Systemic therapy side effects**	**Upset by hair loss**	**Arm symptoms**	**Breast symptoms**	**Endocrine therapy**	**Skin mucosis**	**Endocrine sexual**
*Age*							
≤ 50	46.14 (22.23)	70.75 (44.43)	32.06 (26.13)	14.93 (17.76)	45.34 (22.03)	32.53 (21.49)	38.12 (34.44)
> 50	43.32 (19.87)	61.58 (47.65)	32.93 (20.02)	13.76 (14.27)	39.97 (19.58)	31.54 (15.73)	33.71 (33.75)
*p* value	0.518	0.334	0.856	0.724	0.214	0.800	0.531
*Surgery*							
Yes	46.49 (22.67)	60.52 (47.83)	34.58 (25.80)	15.10 (16.82)	42.81 (21.16)	32.66 (19.20)	36.18 (34.46)
No	41.06 (16.80)	78.86 (39.64)	27.93 (15.87)	12.76 (14.53)	42.60 (20.82)	30.73 (18.23)	35.56 (33.54)
*p* value	0.245	0.071	0.197	0.513	0.963	0.639	0.935
*Menopausal status*							
Pre	44.38 (23.56)	74.38 (42.48)	30.15 (22.57)	14.69 (20.45)	43.65 (22.98)	32.88 (20.74)	28.84 (32.48)
Post	44.92 (20.22)	63.27 (47.20)	33.36 (23.60)	14.24 (14.29)	42.40 (20.29)	31.73 (18.21)	38.68 (34.40)
*p* value	0.912	0.296	0.545	0.905	0.797	0.793	0.211
*Comorbidity*							
Yes	45.67 (19.50)	60.00 (47.27)	32.00 (19.63)	13.67 (15.34)	44.15 (19.74)	33.80 (16.50)	36.47 (37.68)
No	44.12 (22.28)	70.91 (44.93)	32.83 (25.74)	14.87 (16.73)	41.72 (21.90)	30.78 (20.41)	35.63 (31.41)
*p* value	0.726	0.256	0.864	0.719	0.581	0.443	0.906
*HER2 status*							
Yes	41.65 (20.62)	63.53 (46.66)	26.25 (20.51)	14.90 (16.54)	42.00 (23.09)	30.65 (18.71)	33.09 (31.44)
No	46.36 (21.26)	67.73 (45.97)	35.65 (24.06)	14.09 (15.98)	43.12 (19.95)	32.76 (19.00)	37.46 (35.38)
*p* value	0.302	0.676	0.062	0.820	0.806	0.608	0.557
*Breast cancer family history*							
Yes	46.29 (18.82)	64.58 (46.34)	37.83 (27.16)	15.91 (19.85)	45.89 (21.78)	30.66 (20.71)	37.54 (35.01)
No	43.23 (23.22)	68.08 (46.08)	27.02 (17.05)	12.78 (11.02)	39.53 (19.77)	33.46 (16.80)	34.40 (33.24)
*p* value	0.482	0.713	**0.023**	0.346	0.140	0.471	0.655
*Marital status*							
Married	44.67 (20.72)	69.86 (44.85)	32.13 (24.50)	14.82 (17.51)	43.83 (20.77)	32.67 (19.19)	37.02 (34.63)
Divorce	52.50 (22.54)	25.00 (50.00)	47.00 (14.30)	18.75 (10.40)	54.25 (19.61)	22.00 (12.70)	50.00 (19.62)
Widow	45.18 (19.75)	81.81 (34.55)	33.09 (19.22)	10.63 (10.62)	36.18 (18.90)	33.81 (21.38)	36.27 (35.59)
Single	40.85 (28.88)	28.57 (48.79)	26.85 (19.15)	13.00 (9.55)	35.14 (25.71)	28.57 (14.17)	16.71 (28.96)
*p* value	0.857	**0.020**	0.581	0.807	0.337	0.676	0.397
*Stages*							
Stages 1 and 2	44.85 (19.55)	67.62 (46.78)	30.28 (18.76)	14.54 (16.01)	36.51 (21.41)	32.51 (17.84)	34.51 (34.26)
Stages 3 and 4	44.73 (22.05)	65.55 (45.92)	33.76 (25.57)	14.26 (16.27)	46.38 (19.96)	31.78 (19.52)	36.85 (34.10)
*p* value	0.978	0.834	0.485	0.936	**0.030**	0.856	0.749
*Breast cancer medications*							
Taxol	47.64 (20.23)	66.08 (45.64)	34.14 (20.35)	14.87 (16.19)	45.54 (22.60)	32.24 (19.09)	39.21 (35.26)
Gemcitabine	46.57 (6.68)	71.42 (48.79)	54.00 (35.48)	26.00 (31.28)	46.57 (14.45)	43.42 (18.27)	47.57 (36.23)
Doxorubicin	36.80 (21.49)	73.00 (44.26)	16.80 (16.29)	10.80 (8.91)	31.90 (16.59)	20.04 (14.73)	27.38 (29.56)
Others	53.75 (25.61)	75.00 (50.00)	49.75 (34.68)	6.00 (4.00)	42.25 (20.71)	37.50 (27.14)	21.00 (31.69)
Combination	37.33 (26.15)	33.33 (51.63)	35.00 (16.57)	14.00 (11.41)	50.00 (15.84)	41.33 (17.13)	32.00 (35.97)
*p* value	0.233	0.446	**0.001**	0.213	0.105	0.083	0.481

*Note:* Bold *p* values are statistically significant.

^a^This analysis was not conducted because of missing data of patients who did not undergo surgery.

^b^This analysis was not conducted because of missing data concerning sexual enjoyment of divorce, widows, and single patients.

## Data Availability

The data supporting the findings of this study are available from the corresponding author upon reasonable request. All data was collected and analyzed in accordance with ethical guidelines and regulations protecting patient privacy.

## Supporting Information

Supporting Information Additional supporting information can be found online in the Supporting Information section. Supporting Information contains Table S4 with the details of the univariate and multivariate linear regression analysis with parameter estimates for QoL functional scale and global health of EROTC QLQ-C30.
