# Supplementary material for: Quality of Life Assessment of Breast Cancer Patients Undergoing Chemotherapy in Jordan: A Cross-Sectional Study
Source: Int J Breast Cancer. 2025 Mar 1;2025:9936131. doi: 10.1155/ijbc/9936131 (PMC11991791; doi:10.1155/ijbc/9936131)
Supplement: Supporting Information — Additional supporting information can be found online in the Supporting Information section. Supporting Information contains Table S4 with the details of the univariate and multivariate linear regression analysis with parameter estimates for QoL functional scale and global health of EROTC QLQ-C30. [file 9936131.f1.docx]

**Quality of Life Assessment of Breast Cancer Patients Undergoing Chemotherapy in Jordan: A Cross-Sectional Study**

*Sijood janabi ^1^, Lobna Gharaibeh ^2*^, Ibrahim Aldeeb ^3^ , Ali Abuhaliema ^4^*

^1^ Biopharmaceutics and Clinical Pharmacy Department, Faculty of Pharmacy, Al-Ahliyya Amman University, Amman 19328, Jordan. [sijoodjanabi@gmail.com](mailto:sijoodjanabi@gmail.com)

^2^ Biopharmaceutics and Clinical Pharmacy Department, Faculty of Pharmacy, Al-Ahliyya Amman University, Amman 19328, Jordan. [L.gharaibeh@ammanu.edu.jo](mailto:L.gharaibeh@ammanu.edu.jo)

^3^ Faculty of Pharmacy, Zarqa University, Zarqa 13132, Jordan. [ialdeeb@zu.edu.jo](mailto:ialdeeb@zu.edu.jo)

^4^ Biopharmaceutics and Clinical Pharmacy Department, Faculty of Pharmacy, Al-Ahliyya Amman University, Amman 19328, Jordan. [a.abuhaliema@ammanu.edu.jo](mailto:a.abuhaliema@ammanu.edu.jo)

***Corresponding author:**

Lobna Gharaibeh

Biopharmaceutics and Clinical Pharmacy Department, Faculty of Pharmacy, Al-Ahliyya Amman University, Amman 19328, Jordan.

Email address: [l.gharaibeh@ammanu.edu.jo](mailto:l.gharaibeh@ammanu.edu.jo)

ORCID: 0000-0002-7490-5465

Mobile: +962796939977

Table 4. Linear regression model with parameter estimates for QoL functional scale and global health of EROTC QLQ-C30

|  | **Global health Mean/QoL** | | | **Physical Functioning** | | | **Role Functioning** | | | **Emotional Functioning** | | | **Cognitive Functioning** | | | **Social Functioning** | | |
| --- | --- | --- | --- | --- | --- | --- | --- | --- | --- | --- | --- | --- | --- | --- | --- | --- | --- | --- |
|  | **B** | **95%CI** | **p** | **B** | **95%CI** | **p** | **B** | **95%CI** | **p** | **B** | **95%CI** | **p** | **B** | **95%CI** | **p** | **B** | **95%CI** | **p** |
| **Age** | | | | | | | | | | | | | | | | | | |
| <=50 (Reference) |  |  |  |  |  |  |  |  |  |  |  |  |  |  |  |  |  |  |
| >50 | 0.104 | -6.039-13.922 | 0.434 | 0.108 | -7.533-17.452 | 0.432 | 0.164 | -5.608-28.153 | 0.188 | 0.276 | -0.172-32.647 | 0.052 | 0.214 | -4.043-29.995 | 0.133 | 0.074 | -12.204-21.909 | 0.573 |
| **Surgery** | | | | | | | | | | | | | | | | | | |
| Yes (Reference) |  |  |  |  |  |  |  |  |  |  |  |  |  |  |  |  |  |  |
| No | 0.043 | -7.260-10.791 | 0.698 | 0.059 | -8.377-14.218 | 0.608 | -0.042 | -18.366-12.165 | 0.687 | 0.047 | -11.887-17.793 | 0.693 | -0.066 | -19.694-11.087 | 0.579 | 00.039 | -12.678-18.171 | 0.724 |
| **Menopausal status** | | | | | | | | | | | | | | | | | | |
| Pre (Reference) |  |  |  |  |  |  |  |  |  |  |  |  |  |  |  |  |  |  |
| Post | -0.272 | -22.814--.222 | **0.046** | -0.185 | -23.681-4.598 | 0.183 | -0.247 | -38.191-0.022 | **0.050** | -0.080 | -23.831-13.316 | 0.575 | 0.073 | -14.295-24.230 | 0.609 | 0.048 | -15.751-22.861 | 0.715 |
| **Comorbidity** | | | | | | | | | | | | | | | | | | |
| Yes (Reference) |  |  |  |  |  |  |  |  |  |  |  |  |  |  |  |  |  |  |
| No | 0.047 | -7.586-11.196 | 0.703 | 0.093 | -7.420-16.091 | 0.465 | 0.103 | -8.693-23.075 | 0.370 | -0.054 | -18.657-12.225 | 0.680 | 0.025 | -14.484-17.545 | .850 | .087 | -10.262-21.838 | .475 |
| **HER2 Status** | | | | | | | | | | | | | | | | | | |
| Yes (Reference) |  |  |  |  |  |  |  |  |  |  |  |  |  |  |  |  |  |  |
| No | -0.157 | -15.180-2.590 | .162 | -0.005 | -11.368-10.876 | 0.965 | -0.056 | -19.108-10.948 | 0.590 | -0.229 | -28.898-0.320 | 0.055 | 0.034 | -12.98-17.321 | 0.776 | -0.158 | -26.145-4.225 | 0.155 |
| **Family History** | | | | | | | | | | | | | | | | | | |
| Yes (reference) |  |  |  |  |  |  |  |  |  |  |  |  |  |  |  |  |  |  |
| No | -0.011 | -8.485-7.637 | .917 | -.037 | -11.792-8.389 | 0.738 | 0.056 | -9.788**_** 17.481 | 0.576 | 0.103 | -7.192-19.317 | 0.365 | 0.143 | -5.074-22.419 | 0.213 | 0.015 | -12.798-14.756 | 0.888 |
| **Marital Status** | | | | | | | | | | | | | | | | | | |
| Married | 0-.152 | -21.952-8.334 | 0.373 | 0.019 | -17.918-19.991 | 0.914 | 0.209 | -8.599-42.626 | 0.190 | 0.101 | -17.832-31.964 | 0.574 | 0.098 | -18.771-32.873 | 0.588 | -0.079 | -32.035-19.724 | 0.637 |
| Divorce | -0.216 | -44.142-3.399 | 0.092 | -0.104 | -41.659-17.850 | 0.428 | -0.003 | -40.743-39.668 | 0.979 | -0.015 | -41.212-36.957 | 0.914 | 0.013 | -38.636-42.434 | 0.926 | -0.356 | -98.636 - -17.386 | **0.006** |
| Widow | 0.019 | -17.437-19.639 | 0.906 | 0.084 | -17.145-29.265 | 0.605 | 0.394 | 10.993-73.704 | **0.009** | 0.112 | -20.163-40.799 | 0.502 | 0.119 | -20.347-42.877 | 0.480 | 0.096 | -21.801-41.563 | 0.537 |
| Single (reference) |  |  |  |  |  |  |  |  |  |  |  |  |  |  |  |  |  |  |
| **Stages** |  |  |  |  |  |  |  |  |  |  |  |  |  |  |  |  |  |  |
| Stage 1,2 (reference) |  |  |  |  |  |  |  |  |  |  |  |  |  |  |  |  |  |  |
| Stage 3,4 | -0.168 | -15.449-2.261 | 0.142 | -0.149 | -18.212-3.955 | 0.204 | -0.226 | -31.074--1.121 | **0.035** | -0.084 | -19.676-9.441 | 0.486 | -0.073 | -19.703-10.495 | 0.546 | 0.036 | -12.657-17.609 | 0.746 |
| **Medications** | | | | | | | | | | | | | | | | | | |
| Taxol | -0.134 | -22.051-11.717 | 0.544 | -0.006 | -21.420-20.848 | 0.979 | -0.122 | -37.103--37.103 | 0.553 | -0.017 | -28.773-26.748 | 0.942 | 0.279 | -11.555-46.027 | 0.237 | -0.306 | -49.294-8.416 | 0.162 |
| gemcitabine | -0.071 | -27.179-16.923 | 0.645 | -0.108 | -37.107-18.097 | 0.495 | -0.236 | -68.369-6.225 | 0.101 | 0.069 | -28.455-44.058 | 0.670 | 0.314 | -1.209-73.995 | 0.058 | -0.068 | -46.171-29.200 | 0.655 |
| Doxorubicin | 0.030 | -17.113-19.831 | 0.884 | 0.219 | -10.965-35.278 | 0.299 | 0.002 | -31.046-31.439 | 0.990 | -0.080 | -36.045-24.698 | 0.711 | 0.147 | -20.798-42.199 | 0.501 | -0.247 | -51.067-12.070 | 0.223 |
| Others | -0.062 | -30.697-19.083 | 0.644 | -0.079 | -40.250-22.061 | 0.563 | -0.128 | -64.022-20.176 | 0.303 | 0.018 | -38.290-43.560 | 0.898 | 0.112 | -25.500-59.388 | 0.429 | 0.013 | -40.372-44.704 | 0.920 |
| Combination (reference) |  |  |  |  |  |  |  |  |  |  |  |  |  |  |  |  |  |  |
